# 4-(2-Oxa-6-aza­spiro­[3.3]hept-6-yl)­benzo­nitrile

**DOI:** 10.1107/S1600536810012936

**Published:** 2010-04-28

**Authors:** Jian Li, Ting Xu, Peng-Bo Wu, Jing Lu, Yao Tang

**Affiliations:** a Department of Pharmacy, West China Hospital of Sichuan University, Chengdu 610041, People’s Republic of China

## Abstract

In the title compound, C_12_H_12_N_2_O, the azetidine ring (r.m.s. deviation = 0.021 Å) and the oxetane ring (r.m.s. deviation = 0.014 Å) are nearly perpendicular to each other [dihedral angle = 89.7 (1)°]. The azetidine ring is twisted out of the plane of the benzene ring by 18.3 (1)°. In the crystal structure, mol­ecules are linked to form chains along the *c* axis by C—H⋯O hydrogen bonds.

## Related literature

The title compound is a key inter­mediate to synthesize (pyrrolo[3,4-*c*]pyrazol-3-yl)benzamide derivatives. For the anti-tumor effect of these derivatives, see: Fancelli *et al.* (2005 ▶).
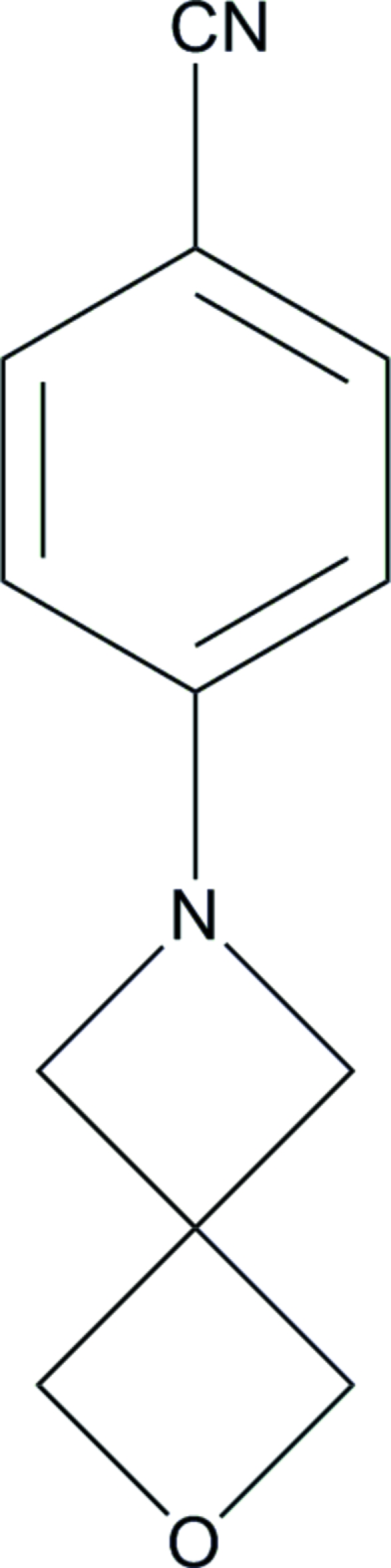


## Experimental

### 

#### Crystal data

C_12_H_12_N_2_O
*M* *_r_* = 200.24
Monoclinic, 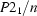

*a* = 9.484 (4) Å
*b* = 11.033 (4) Å
*c* = 10.419 (4) Å
β = 113.186 (5)°
*V* = 1002.2 (7) Å^3^
*Z* = 4
Mo *K*α radiation
μ = 0.09 mm^−1^
*T* = 113 K
0.60 × 0.60 × 0.27 mm

#### Data collection

Rigaku AFC10/Saturn 724-Plus diffractometer
7662 measured reflections
2234 independent reflections
1872 reflections with *I* > 2σ(*I*)
*R*_int_ = 0.029

#### Refinement

*R*[*F* ^2^ > 2σ(*F* ^2^)] = 0.041
*wR*(*F* ^2^) = 0.099
*S* = 1.00
2234 reflections
136 parameters
H-atom parameters constrained
Δρ_max_ = 0.30 e Å^−3^
Δρ_min_ = −0.16 e Å^−3^

### 

Data collection: *CrystalClear* (Rigaku, 2008 ▶); cell refinement: *CrystalClear*; data reduction: *CrystalClear*; program(s) used to solve structure: *SHELXS97* (Sheldrick, 2008 ▶); program(s) used to refine structure: *SHELXL97* (Sheldrick, 2008 ▶); molecular graphics: *PLATON* (Spek, 2009 ▶); software used to prepare material for publication: *SHELXL97*.

## Supplementary Material

Crystal structure: contains datablocks global, I. DOI: 10.1107/S1600536810012936/ci5062sup1.cif

Structure factors: contains datablocks I. DOI: 10.1107/S1600536810012936/ci5062Isup2.hkl

Additional supplementary materials:  crystallographic information; 3D view; checkCIF report

## Figures and Tables

**Table 1:** Hydrogen-bond geometry (Å, °)

*D*—H⋯*A*	*D*—H	H⋯*A*	*D*⋯*A*	*D*—H⋯*A*
C4—H4⋯O1^i^	0.95	2.47	3.371 (2)	159

Symmetry code: (i) 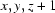.

## supplementary crystallographic information

### Experimental

A DMSO solution of 2-oxa-6-azaspiro[3,3]heptane (0.99 g, 0.01 mol) with 4-fluorobenzonitrile (1.21 g, 0.01 mol) was heated to reflux for 5 h, then water (100 ml) was added into the solution. The mixture was extracted with CH_2_Cl_2_. Then the solvent was removed to give a red powder. Single crystals were obtained from a CH_2_Cl_2_ solution after 3 days.

### Refinement

H atoms were placed in the calculated positions (C—H = 0.95–0.99 Å) and refined using a riding model with *U*_iso_(H) = 1.2*U*_eq_(C).

### Crystal data

**Table:**

C_12_H_12_N_2_O	*F*(000) = 424
*M**_r_* = 200.24	*D*_x_ = 1.327 Mg m^−^^3^
Monoclinic, *P*2_1_/*n*	Mo *K*α radiation, λ = 0.71073 Å
*a* = 9.484 (4) Å	Cell parameters from 2956 reflections
*b* = 11.033 (4) Å	θ = 3.1–27.5°
*c* = 10.419 (4) Å	µ = 0.09 mm^−^^1^
β = 113.186 (5)°	*T* = 113 K
*V* = 1002.2 (7) Å^3^	Prism, colourless
*Z* = 4	0.60 × 0.60 × 0.27 mm

### Data collection

**Table:**

Rigaku AFC10/Saturn 724-Plus diffractometer	1872 reflections with *I* > 2σ(*I*)
Radiation source: rotating anode	*R*_int_ = 0.029
graphite	θ_max_ = 27.5°, θ_min_ = 3.1°
Detector resolution: 28.5714 pixels mm^-1^	*h* = −12→12
φ and ω scans	*k* = −14→11
7662 measured reflections	*l* = −12→12
2234 independent reflections	

### Refinement

**Table:**

Refinement on *F*^2^	Primary atom site location: structure-invariant direct methods
Least-squares matrix: full	Secondary atom site location: difference Fourier map
*R*[*F*^2^ > 2σ(*F*^2^)] = 0.041	Hydrogen site location: inferred from neighbouring sites
*wR*(*F*^2^) = 0.099	H-atom parameters constrained
*S* = 1.00	*w* = 1/[σ^2^(*F*_o_^2^) + (0.0456*P*)^2^ + 0.36*P*] where *P* = (*F*_o_^2^ + 2*F*_c_^2^)/3
2234 reflections	(Δ/σ)_max_ = 0.001
136 parameters	Δρ_max_ = 0.30 e Å^−^^3^
0 restraints	Δρ_min_ = −0.16 e Å^−^^3^

### Special details

**Table:**

Geometry. All esds (except the esd in the dihedral angle between two l.s. planes) are estimated using the full covariance matrix. The cell esds are taken into account individually in the estimation of esds in distances, angles and torsion angles; correlations between esds in cell parameters are only used when they are defined by crystal symmetry. An approximate (isotropic) treatment of cell esds is used for estimating esds involving l.s. planes.
Refinement. Refinement of *F*^2^ against ALL reflections. The weighted *R*-factor wR and goodness of fit *S* are based on *F*^2^, conventional *R*-factors *R* are based on *F*, with *F* set to zero for negative *F*^2^. The threshold expression of *F*^2^ > σ(*F*^2^) is used only for calculating *R*-factors(gt) etc. and is not relevant to the choice of reflections for refinement. *R*-factors based on *F*^2^ are statistically about twice as large as those based on *F*, and *R*- factors based on ALL data will be even larger.

### Fractional atomic coordinates and isotropic or equivalent isotropic displacement parameters (Å^2^)

**Table:**

	*x*	*y*	*z*	*U*_iso_*/*U*_eq_	
O1	0.53098 (12)	0.21116 (9)	−0.00129 (10)	0.0272 (3)	
N1	0.81838 (14)	0.46062 (12)	1.07793 (13)	0.0275 (3)	
N2	0.58259 (13)	0.37004 (10)	0.38573 (11)	0.0186 (3)	
C1	0.74599 (15)	0.47583 (12)	0.59326 (14)	0.0178 (3)	
H1	0.7933	0.5192	0.5421	0.021*	
C2	0.79050 (15)	0.49646 (12)	0.73352 (14)	0.0186 (3)	
H2	0.8679	0.5547	0.7788	0.022*	
C3	0.72270 (15)	0.43219 (12)	0.81086 (14)	0.0177 (3)	
C4	0.60810 (15)	0.34638 (12)	0.74350 (14)	0.0183 (3)	
H4	0.5624	0.3022	0.7954	0.022*	
C5	0.56183 (15)	0.32601 (12)	0.60278 (14)	0.0178 (3)	
H5	0.4835	0.2683	0.5577	0.021*	
C6	0.62984 (14)	0.39026 (12)	0.52463 (13)	0.0165 (3)	
C7	0.49702 (15)	0.26581 (12)	0.30534 (14)	0.0178 (3)	
H7A	0.3842	0.2751	0.2702	0.021*	
H7B	0.5303	0.1871	0.3534	0.021*	
C8	0.56407 (14)	0.29329 (12)	0.19522 (14)	0.0167 (3)	
C9	0.65782 (15)	0.39702 (12)	0.28976 (13)	0.0177 (3)	
H9A	0.7699	0.3827	0.3302	0.021*	
H9B	0.6331	0.4782	0.2461	0.021*	
C10	0.45979 (16)	0.31151 (12)	0.04116 (14)	0.0204 (3)	
H10A	0.3499	0.2979	0.0208	0.024*	
H10B	0.4749	0.3907	0.0037	0.024*	
C11	0.63751 (16)	0.19237 (13)	0.14221 (14)	0.0226 (3)	
H11A	0.7455	0.2092	0.1575	0.027*	
H11B	0.6281	0.1114	0.1790	0.027*	
C12	0.77569 (15)	0.44870 (12)	0.95891 (15)	0.0202 (3)	

### Atomic displacement parameters (Å^2^)

**Table:**

	*U*^11^	*U*^22^	*U*^33^	*U*^12^	*U*^13^	*U*^23^
O1	0.0353 (6)	0.0266 (6)	0.0202 (5)	0.0027 (5)	0.0113 (5)	−0.0041 (4)
N1	0.0257 (7)	0.0323 (7)	0.0231 (7)	−0.0015 (5)	0.0082 (5)	−0.0038 (6)
N2	0.0203 (6)	0.0187 (6)	0.0180 (6)	−0.0073 (4)	0.0086 (5)	−0.0027 (5)
C1	0.0179 (6)	0.0147 (6)	0.0217 (7)	−0.0007 (5)	0.0086 (5)	0.0028 (5)
C2	0.0156 (6)	0.0151 (6)	0.0223 (7)	0.0007 (5)	0.0043 (5)	−0.0017 (5)
C3	0.0165 (6)	0.0176 (6)	0.0177 (7)	0.0041 (5)	0.0054 (5)	−0.0004 (5)
C4	0.0177 (6)	0.0181 (6)	0.0212 (7)	0.0025 (5)	0.0098 (5)	0.0022 (5)
C5	0.0157 (6)	0.0163 (6)	0.0212 (7)	−0.0017 (5)	0.0069 (5)	−0.0012 (5)
C6	0.0151 (6)	0.0147 (6)	0.0188 (7)	0.0023 (5)	0.0058 (5)	0.0008 (5)
C7	0.0184 (6)	0.0157 (6)	0.0195 (7)	−0.0029 (5)	0.0076 (5)	−0.0014 (5)
C8	0.0161 (6)	0.0149 (6)	0.0193 (7)	0.0004 (5)	0.0071 (5)	0.0002 (5)
C9	0.0171 (6)	0.0186 (6)	0.0172 (6)	−0.0020 (5)	0.0065 (5)	0.0004 (5)
C10	0.0210 (7)	0.0184 (6)	0.0195 (7)	−0.0010 (5)	0.0057 (5)	−0.0002 (6)
C11	0.0257 (7)	0.0215 (7)	0.0227 (7)	0.0046 (6)	0.0117 (6)	0.0016 (6)
C12	0.0175 (6)	0.0195 (6)	0.0235 (8)	0.0024 (5)	0.0079 (5)	−0.0010 (6)

### Geometric parameters (Å, °)

**Table:**

O1—C11	1.4525 (17)	C5—C6	1.4125 (19)
O1—C10	1.4534 (17)	C5—H5	0.95
N1—C12	1.1504 (18)	C7—C8	1.5451 (18)
N2—C6	1.3544 (17)	C7—H7A	0.99
N2—C7	1.4653 (17)	C7—H7B	0.99
N2—C9	1.4695 (17)	C8—C11	1.5274 (18)
C1—C2	1.3711 (19)	C8—C10	1.5314 (18)
C1—C6	1.4127 (18)	C8—C9	1.5431 (18)
C1—H1	0.95	C9—H9A	0.99
C2—C3	1.4049 (19)	C9—H9B	0.99
C2—H2	0.95	C10—H10A	0.99
C3—C4	1.4034 (19)	C10—H10B	0.99
C3—C12	1.4335 (19)	C11—H11A	0.99
C4—C5	1.3734 (19)	C11—H11B	0.99
C4—H4	0.95		
			
C11—O1—C10	90.79 (9)	H7A—C7—H7B	111.1
C6—N2—C7	128.02 (11)	C11—C8—C10	85.12 (10)
C6—N2—C9	130.44 (11)	C11—C8—C9	122.78 (11)
C7—N2—C9	94.47 (10)	C10—C8—C9	122.94 (11)
C2—C1—C6	120.13 (12)	C11—C8—C7	120.34 (11)
C2—C1—H1	119.9	C10—C8—C7	121.30 (11)
C6—C1—H1	119.9	C9—C8—C7	88.49 (10)
C1—C2—C3	120.61 (12)	N2—C9—C8	88.40 (10)
C1—C2—H2	119.7	N2—C9—H9A	113.9
C3—C2—H2	119.7	C8—C9—H9A	113.9
C4—C3—C2	119.53 (12)	N2—C9—H9B	113.9
C4—C3—C12	119.89 (12)	C8—C9—H9B	113.9
C2—C3—C12	120.49 (12)	H9A—C9—H9B	111.1
C5—C4—C3	120.22 (12)	O1—C10—C8	91.91 (10)
C5—C4—H4	119.9	O1—C10—H10A	113.3
C3—C4—H4	119.9	C8—C10—H10A	113.3
C4—C5—C6	120.45 (12)	O1—C10—H10B	113.3
C4—C5—H5	119.8	C8—C10—H10B	113.3
C6—C5—H5	119.8	H10A—C10—H10B	110.6
N2—C6—C5	119.95 (12)	O1—C11—C8	92.11 (10)
N2—C6—C1	121.00 (12)	O1—C11—H11A	113.3
C5—C6—C1	119.05 (12)	C8—C11—H11A	113.3
N2—C7—C8	88.47 (10)	O1—C11—H11B	113.3
N2—C7—H7A	113.9	C8—C11—H11B	113.3
C8—C7—H7A	113.9	H11A—C11—H11B	110.6
N2—C7—H7B	113.9	N1—C12—C3	179.26 (15)
C8—C7—H7B	113.9		
			
C6—C1—C2—C3	0.67 (19)	N2—C7—C8—C11	130.78 (12)
C1—C2—C3—C4	−0.17 (18)	N2—C7—C8—C10	−125.31 (12)
C1—C2—C3—C12	176.30 (12)	N2—C7—C8—C9	3.04 (9)
C2—C3—C4—C5	−0.45 (19)	C6—N2—C9—C8	154.66 (13)
C12—C3—C4—C5	−176.95 (12)	C7—N2—C9—C8	3.21 (10)
C3—C4—C5—C6	0.57 (19)	C11—C8—C9—N2	−128.76 (12)
C7—N2—C6—C5	−18.4 (2)	C10—C8—C9—N2	123.99 (12)
C9—N2—C6—C5	−161.23 (13)	C7—C8—C9—N2	−3.04 (9)
C7—N2—C6—C1	162.08 (12)	C11—O1—C10—C8	−2.22 (10)
C9—N2—C6—C1	19.3 (2)	C11—C8—C10—O1	2.12 (10)
C4—C5—C6—N2	−179.56 (12)	C9—C8—C10—O1	128.42 (12)
C4—C5—C6—C1	−0.07 (19)	C7—C8—C10—O1	−120.66 (12)
C2—C1—C6—N2	178.93 (12)	C10—O1—C11—C8	2.22 (10)
C2—C1—C6—C5	−0.55 (19)	C10—C8—C11—O1	−2.12 (10)
C6—N2—C7—C8	−155.71 (13)	C9—C8—C11—O1	−128.56 (12)
C9—N2—C7—C8	−3.21 (10)	C7—C8—C11—O1	121.54 (12)

### Hydrogen-bond geometry (Å, °)

**Table:**

*D*—H···*A*	*D*—H	H···*A*	*D*···*A*	*D*—H···*A*
C4—H4···O1^i^	0.95	2.47	3.371 (2)	159

Symmetry codes: (i) *x*, *y*, *z*+1.
